# Implementation of a patient-centered remote wound monitoring system for management of diabetic foot ulcers

**DOI:** 10.3389/fendo.2023.1157518

**Published:** 2023-05-24

**Authors:** Alana C. Keegan, Sanuja Bose, Katherine M. McDermott, Midori P. Starks White, David P. Stonko, Danielle Jeddah, Eilat Lev-Ari, Joanna Rutkowski, Ronald Sherman, Christopher J. Abularrage, Elizabeth Selvin, Caitlin W. Hicks

**Affiliations:** ^1^ Department of Surgery, Sinai Hospital of Baltimore, Baltimore, MD, United States; ^2^ Division of Vascular Surgery and Endovascular Therapy, Johns Hopkins University, Baltimore, MD, United States; ^3^ Department of Clinical Development, Healthy.io Ltd., Tel Aviv, Israel; ^4^ Department of Epidemiology, Johns Hopkins School of Public Health, Baltimore, MD, United States

**Keywords:** diabetic foot ulcer (DFU), diabetes, smartphone application (app), telemedicine, technology, smartphone, diabetic foot

## Abstract

**Background:**

Regular clinical assessment is critical to optimize lower extremity wound healing. However, family and work obligations, socioeconomic, transportation, and time barriers often limit patient follow-up. We assessed the feasibility of a novel, patient-centered, remote wound management system (Healthy.io Minuteful for Wound Digital Management System) for the surveillance of lower extremity wounds.

**Methods:**

We enrolled 25 patients from our outpatient multidisciplinary limb preservation clinic with a diabetic foot ulcer, who had undergone revascularization and podiatric interventions prior to enrollment. Patients and their caregivers were instructed on how to use the digital management system and asked to perform one at-home wound scan per week for a total of 8 weeks using a smartphone application. We collected prospective data on patient engagement, smartphone app useability, and patient satisfaction.

**Results:**

Twenty-five patients (mean age 65.5 ± 13.7 years, 60.0% male, 52.0% Black) were enrolled over 3 months. Mean baseline wound area was 18.0 ± 15.2 cm^2^, 24.0% of patients were recovering from osteomyelitis, and post-surgical WiFi stage was 1 in 24.0%, 2 in 40.0%, 3 in 28.0%, and 4 in 8.00% of patients. We provided a smartphone to 28.0% of patients who did not have access to one that was compatible with the technology. Wound scans were obtained by patients (40.0%) and caregivers (60.0%). Overall, 179 wound scans were submitted through the app. The mean number of wound scans acquired per patient was 0.72 ± 0.63 per week, for a total mean of 5.80 ± 5.30 scans over the course of 8 weeks. Use of the digital wound management system triggered an early change in wound management for 36.0% of patients. Patient satisfaction was high; 94.0% of patients reported the system was useful.

**Conclusion:**

The Healthy.io Minuteful for Wound Digital Management System is a feasible means of remote wound monitoring for use by patients and/or their caregivers.

## Introduction

Chronic lower extremity wounds are a major source of global morbidity, disability, and healthcare utilization (1–3). Diabetic foot ulcers (DFU) represent an increasingly common and difficult to treat subset of lower extremity wounds (2, 4). In the United States, diabetes affects 37.3 million persons, of whom 19% to 33% will develop a DFU during their lifetime (5, 6). Complications of DFU are common and morbid, including up to a 60% occurrence of diabetic foot infection and a 15% to 20% risk of subsequent lower extremity amputation (4). Both incident DFU and poor healing disproportionately affect socioeconomically vulnerable populations, persons with complex medical needs, and/or persons with limited access to high-quality wound care (7).

In-person multidisciplinary diabetic foot and wound management is standard of care for the treatment of DFU (8, 9). However, the model of multidisciplinary care typically requires frequent in-person wound assessments, which may not be achievable for patients due to numerous barriers. Patients with DFU and their caregivers consistently identify time constraints (e.g., difficulty finding available appointment times, conflicts with occupational and care-giving responsibilities), financial insecurity, mobility deficits, and lack of access to safe transportation as barriers to accessing treatment (10, 11). Remote wound care offers a potential approach to overcoming these barriers.

In response to the COVID-19 pandemic, the use of telemedicine has expanded exponentially in the United States (12) Telemedicine strategies have been applied to the management of DFU with mixed results (13). Patients and physicians have expressed enthusiasm for remote wound monitoring solutions, but most current systems rely on trained healthcare providers (e.g., home care nurses) or non-expert clinicians in the home for execution (14, 15). There is a paucity of data on the feasibility, compliance, and outcomes of a remote wound monitoring system that relies on patients and their caregivers to perform their own wound scans.

The Minuteful for Wound Digital Management System (Healthy.io, Tel Aviv, Israel) is a novel, remote wound monitoring system that captures wound measurements and analyzes tissue distribution in real-time through use of a smartphone application. Use of this digital management system by clinicians has been shown to be successful in non-US healthcare settings such as England (16), but a newer patient-facing version of the technology has recently been developed. We conducted a pilot study of patients with DFU to assess patient engagement, reliability, and satisfaction with the Minuteful for Wound Digital Management System.

## Methods

### Patient population

We enrolled 25 patients who presented to the Johns Hopkins Hospital multidisciplinary diabetic limb preservation clinic with an active DFU between July 1 and November 30, 2022. Patients were considered for enrollment in the study if they were proficient in English, ≥18 years of age, had an active DFU, had completed any planned revascularization and/or wound debridement procedures, and were willing and able to use a smartphone to capture weekly wound scans for an 8-week study period. For patients with multiple wounds, the largest wound that was accessible for imaging was designated to be monitored using the device throughout the study. Patients were excluded from the study if the wound was too large to capture in a single wound scan, if the wound was in a location that was not accessible to the patient or their caregiver, or if they were unable to operate the smartphone application. Patients who wished to participate but did not have access to a smartphone were loaned a smartphone with the app pre-installed for the duration of their participation in the study.

The Johns Hopkins University Institutional Review Board approved the study, and all patients provided written informed consent to participate.

### Minuteful for wound digital management system

**Figure 1:**
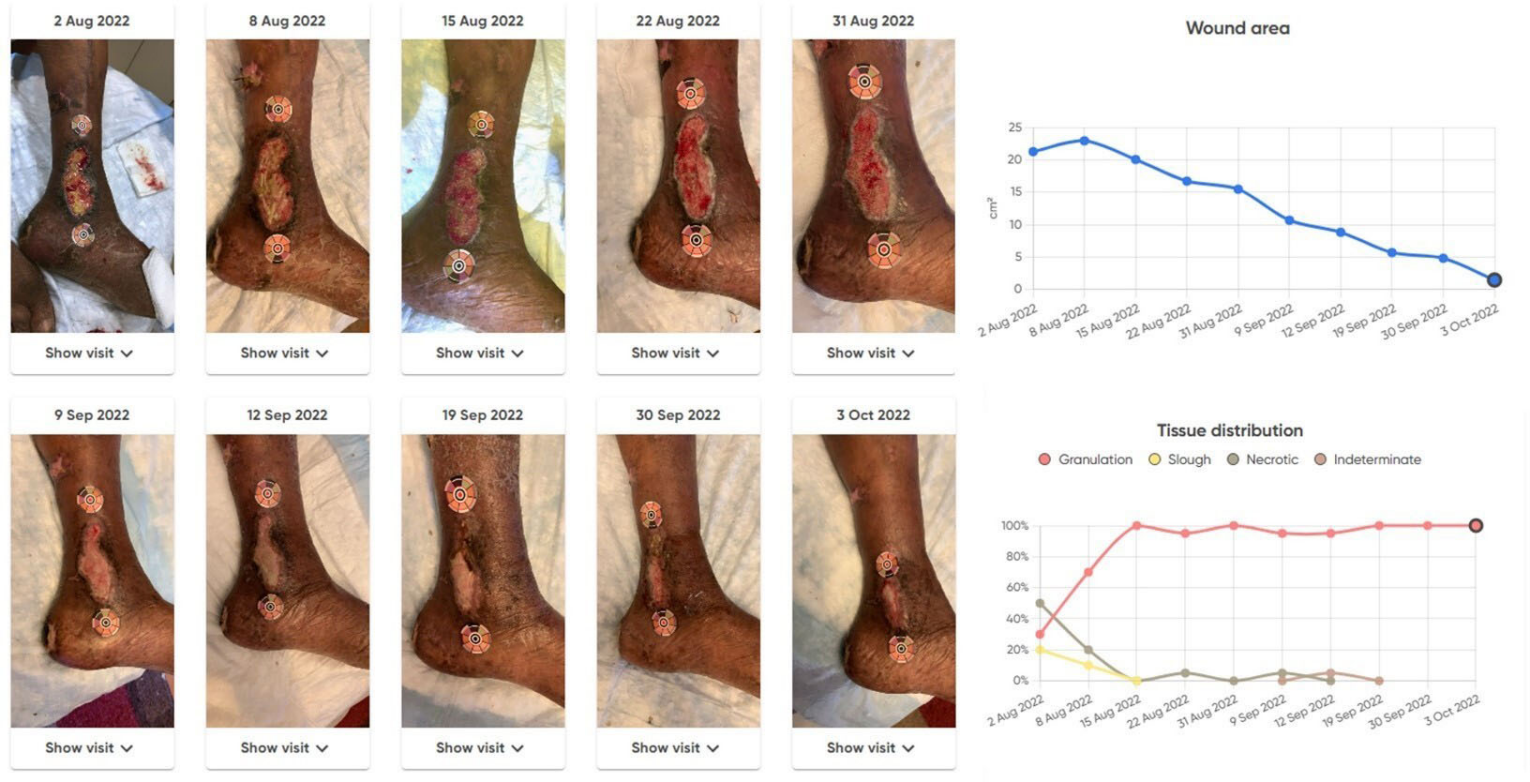
Example wound snapshots showing wound progression over the 8-week study period, along with associated wound area and tissue distribution plots, as provided by the Healthy.io Minuteful for Wound Digital Management System.

**Figure 2:**
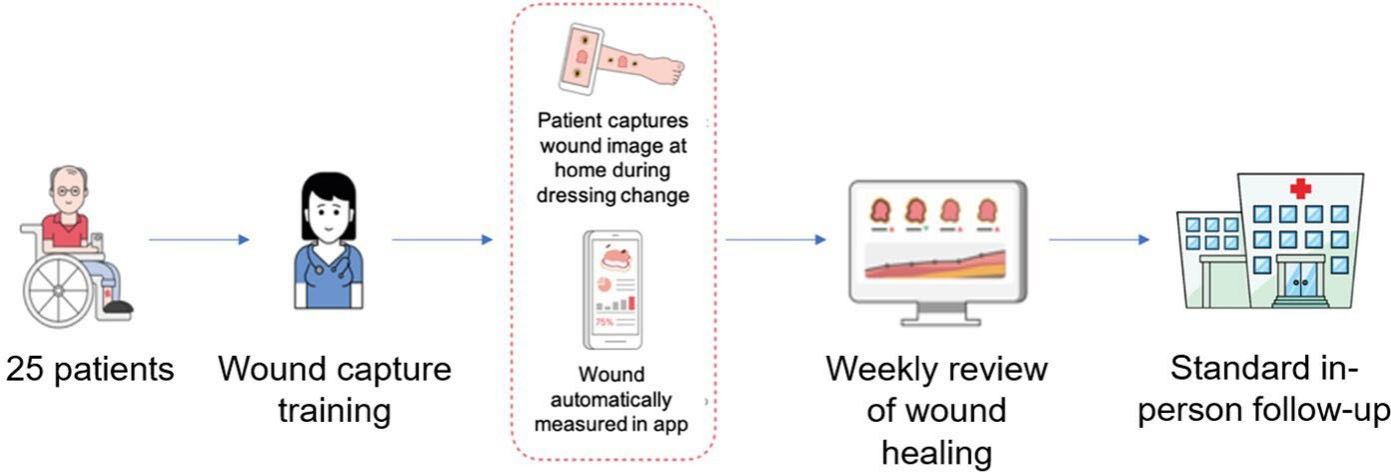
Overview of feasibility study design.

The Minuteful for Wound Digital Management System (Healthy.io, Tel Aviv Israel) consists of dedicated calibration markers (stickers), a smartphone application (Minuteful for Wound app), and a web-based Portal (Minuteful for Wound Portal) that turns any smart mobile device into a wound care management tool (**Figure 1**). The use of calibration markers helps the application identify the wound area and controls for different lighting conditions and camera types. The Minuteful for Wound app guides patients through the process of collecting clinical data and capturing scans of their wound using the embedded smartphone camera. The captured scan is transferred to a cloud-based server, where a set of distinct algorithms is used to analyze and translate it into a set of measurements for each wound. The measurements are securely displayed in the cloud-based Minuteful for Wound Portal to assist healthcare professionals in managing and monitoring the wound healing process (**Figure 2**).

### Study protocol

Following informed consent, the patients and their caregivers were instructed how to download and log into the Minuteful for Wound app on their smartphone. The primary user (patient or caregiver) was then provided with a box of calibration stickers specifically for use with the Minuteful for Wound app and taught how to apply the stickers and scan the wound. The primary user was given the opportunity to ask questions and practice scanning the wound and, once they were proficient, completed the first scan for upload in the clinic. Written information about the study and use of the application, including a user manual and a brochure, were also provided to the patient and their caregiver.

Once trained to use the app, the primary user was asked to obtain weekly at-home wound scans during regular dressing changes. Users were asked to capture a minimum of one wound scan per week to allow for flexibility in scanning, but were encouraged to capture scans with each dressing change when possible. The quality of the scans was standardized using in-app boundary conditions, an algorithmic mechanism that enables results to be presented to the clinicians in the Portal. In cases where the environmental conditions did not meet the device’s prerequisites (e.g., not enough motion during the scan or it was blurry, the lighting was too bright or too dark, or the calibration markers did not remain in the camera field for the entire scan), the patient would be prompted by the app to re-perform the scan. All remotely collected assessments were securely transmitted to the HIPAA-compliant Minuteful for Wound Portal for review by the study team. All wound assessments were reviewed by members of the study team (consisting of a vascular surgeon, surgical podiatrist, and general surgery resident) once per week to assess progress. A weekly wound update was then provided to the patient by phone, and patients with concern for clinically stagnating wounds were asked to visit the clinic for an in-person assessment within the next week. Primary users who did not complete a weekly wound scan were called by the study team with a reminder. A Healthy.io engagement team was available to support the primary user in completing remote assessments as needed. A technology support hotline was available from 8am to 6pm EST on Mondays through Fridays to provide live phone support for app use. At the end of the 8-week study period primary users were asked to complete a useability and satisfaction survey to assess ease of use and usefulness of the Minuteful for Wound Digital Management System. The survey was developed using a mixed Likert scale and open-ended question design through iterative processing by the study team (**Supplementary Table 1**).

### Patient data

We captured age, sex, race, ethnicity, insurance status, area deprivation index and comorbidities for each patient through direct review and abstraction of the electronic medical records. Area deprivation index, which is a comprehensive measure of neighborhood socioeconomic deprivation, was calculated using each patient’s complete address and Neighborhood Atlas mapping (17, 18). Hypertension was defined as systolic blood pressure >160 mmHg on 3 separate visits or current use of antihypertensive medications. Hyperlipidemia was defined as a cholesterol level >200mg/dL, LDL level >130 mg/dL, or use of cholesterol-lowering medications. Coronary artery disease was defined as a documented history of myocardial infarction or previous coronary revascularization. Congestive heart failure was defined based on a documented diagnosis, echocardiogram findings, or the Framingham criteria (19). Peripheral artery disease was defined as an ankle-brachial index (ABI) of ≤0.8, toe pressure of <70 mmHg, or a history of a revascularization procedure on the affected limb. Chronic kidney disease was defined as an eGFR of <90 mL/min/1.73m².

The wound characteristics for each patient were documented by study staff at the time of study enrollment including wound location, size, vascular studies, Wound, Ischemia and foot Infection (WIfI) score (20), and previous revascularization and podiatric surgical interventions. WIfI classification was assigned based on post-revascularization and wound debridement characteristics to determine wound stage at the time of enrollment.

### Study outcomes

The primary outcomes were patient engagement and satisfaction with the Minuteful for Wound Digital Management System. Patient engagement was determined by the recorded number of successful scans performed over the study period. ‘Optimally engaged patients’ were defined as patients completing 100% of study scans (equivalent to at least one scan every week). ‘Highly engaged patients’ were defined as patients meeting a threshold of 75% to 99% of study scans (equivalent to at least one scan every other week and a half). ‘Engaged patients’ were defined as patients meeting a predefined threshold of 50% to 74% of study scans (equivalent to at least one scan every other week, which is equivalent to the expected frequency for standard in-person wound care visits). ‘Not engaged patients’ were defined as patients completing 25% to 49% (equivalent to at least one scan per month). Patients who completed <25% of wound scans (i.e. <2 scans over 8 weeks) were considered to be study failures.

Patient and caregiver satisfaction with the application was determined by survey responses to questions about ease of use and overall usefulness. A patient was determined to be satisfied if they provided a response of 4 or 5 (“Agree” or “Strongly Agree,” respectively) on the Likert Scale.

Secondary outcomes were the proportion of scans that led to a change in patient management, including changes to wound care plan or a change in planned next in-person visit; number of reminder phone calls made to patients; change in wound area from study enrollment to study completion; and the proportion of patients who achieved wound healing. To evaluate the scanning experience of the patients, the number of boundary condition alerts and scan attempts were recorded for each patient assessment.

### Statistical analysis

Baseline patient demographics, comorbidities, wound information, and study outcomes were tabulated and reported using means (standard deviations) or percent (N) as appropriate. Change in wound area over the course of the study was compared using paired t-tests, with P<0.05 denoting statistical significance. Qualitative data collected from open-ended questions on the patient survey were analyzed by two reviewers who used open coding, resolved discrepancies with triangulation, and applied thematic analysis.

## Results

### Patient cohort

We enrolled 25 patients over the month study period. Mean age was 65.5 (SD, 13.7) years, 60.0% of our participants were male, and 52.0% self-identified as non-Hispanic Black adults. The most common comorbidities were hypertension (68.0%), chronic kidney disease (68.0%), and peripheral artery disease (56.0%) (**Table 1**).

**Table 1:** Baseline characteristics of patients included in the feasibility study.

Characteristic	Overall % (N=25)
Age, years (SD)	65.5 (13.6)
Female sex	40.0% (N=10)
Race/Ethnicity	
Non-Hispanic Black	52.0% (N=13)
Non-Hispanic white	48.0% (N=12)
Insurance Status	
Medicare	76.0% (N=19)
Medicaid	16.0% (N=4)
Private/Self Pay/Other	8.00% (N=2)
Other	4.00% (N=1)
Area deprivation index	
Quartile 1 (least deprived)	32.0% (N=8)
Quartile 2	56.0% (N=14)
Quartile 3	4.00% (N=1)
Quartile 4 (most deprived)	8.00% (N=2)
Functional Status	
Independent	72.0% (N=18)
Partially dependent	28.0% (N=7)
Diabetes type	
No medications	32.0% (N=8)
Type 1	NA (N=0)
Type 2 on oral medications	24.0% (N=6)
Type 2 on insulin	44.0% (N=11)
Comorbidities	
Hypertension	68.0% (N=17)
Dyslipidemia	16.0% (N=4)
Coronary artery disease	24.0% (N=6)
Congestive heart failure	12.0% (N=3)
Peripheral artery disease	56.0% (N=14)
Chronic kidney disease	68.0% (N=17)
Dialysis	16.0% (N=4)
Smoking status	
Current	NA (N=0)
Former	72.0% (N=18)
Never	28.0% (N=7)

NA, Not Applicable.

There were a wide variety of wound locations (**Table 2**). Mean wound area at baseline was 18.0 cm² (SD, 15.2), 36.0% of wounds were severe (WIfI stage 3 or 4), 64.0% of patients had undergone lower extremity revascularization, and 80.0% had undergone surgical debridement prior to enrollment. Home health was involved in the care of 60.0% of patients, and a wide range of wound dressing treatment strategies were used (**Table 2**).

**Table 2:** Baseline wound characteristics and related surgical procedures of the study population.

Characteristic	Overall % (N=25)
Wound location	
Lateral/forefoot	44.0% (11)
Lower leg/ankle	32.0% (8)
Heel	12.0% (3)
Plantar foot	8.00% (2)
Toe	4.00% (1)
Wound area, mean cm² ± SD	18.0 ± 15.2
Osteomyelitis	24.0% (6)
WIfI Classification	
1	24.0% (6)
2	40.0% (10)
3	28.0% (7)
4	8.00% (2)
Toe pressure, mean mmHg ± SD (N= 17)	79.6 ± 45.0
Ankle Brachial Index, mean ± SD (N= 16)	1.0 ± 0.2
Related revascularization procedure	
Endovascular	32.0% (8)
Open	32.0% (8)
No. revascularization procedures, mean ± SD	1.1 ± 1.5
Podiatric interventions of the affected limb*	
None	20.0% (5)
Bone resection/debridement	56.0% (14)
Biologic coverage	48.0% (12)
Minor amputation	40.0% (10)
Skin graft	16.0% (4)
Home care	60.0% (15)
Wound dressing type	
Collagen	24.0% (6)
Negative pressure wound therapy	16.0% (4)
Wound hydration	24.0% (6)
Enzymatic debridement	12.0% (3)
Filler	24.0% (6)

WIfI, Wound, Ischemia, and foot Infection.

*Sum equals greater than 100% because some patients received more than one podiatric intervention.

### Study participation

The primary user was a caregiver in 60.0% of cases and the patient in 40.0%. Home health was not involved in wound scanning for this feasibility study. Twenty-eight percent of users borrowed a smartphone for the purposes of study participation.

Overall, patients submitted a mean number of 5.80 (SD, 5.30) wound scans over the total 8-week study period, equal to a mean of 0.72 (SD, 0.63) wound scans per week. Patients received a mean of 2.28 (SD, 2.25) reminder phone calls to submit wound scans from the study team during the study period (**Table 3**). Patients made or received a mean of 6.32 (SD, 5.18) technical calls from the Healthy.io technical team.

**Table 3:** Summary of Healthy.io Minuteful for Wound smartphone app useability.

Characteristic	Overall % (N=25)
Borrowed smartphone	28.0% (N=7)
Did not have smartphone	NA (N=0)
Personal smartphone not compatible	28.0% (N=7)
Primary wound scanner	
Patient	40.0% (N=10)
Caregiver	60.0% (N=15)
No. phone calls per patient during study period, mean (SD)	
Reminder calls from study team	2.28 (2.25)
Technical calls from Healthy.io team	6.32 (5.18)
No. scans completed overall per patient, mean (SD)	5.80 (5.30)
No. scans per week per patient, mean (SD)	0.72 (0.63)

NA, Not Applicable.

### Clinical study outcomes

App engagement was variable, with 20.0% of patients completing 100% of the weekly wound scans (optimally engaged), 28.0% completing 75-99% of weekly wound scans (highly engaged), 12.0% completing 50-74% of weekly wound scans (engaged), and 28.0% completing <25% weekly wounds scans (i.e., study failure) (**Table 4**). Study failures were investigated and classified as communication difficulties in 16.0% of patients and lack of caregiver availability for assistance with the scans in 12.0% of patients. Overall, 21/25 (84.0%) patients completed at least one in-home wound scan.

**Table 4:** Patient outcomes related to Healthy.io Minuteful for Wound smartphone app use.

Outcome	Overall % (N= 25)
Study engagement	
Optimally engaged (100% weekly scans)	20.0% (N=5)
Highly engaged (≥ 75% weekly scans)	28.0% (N=7)
Engaged (≥ 50% weekly scans)	12.0% (N=3)
Not engaged (≥ 25% weekly scans)	12.0% (N=3)
Study failure (< 25% weekly scans)	28.0% (N=7)
Reasons for study failure	
Communication/information flow	16.0% (N=4)
Caregiver availability	12.0% (N=3)
Change in management as a result of scan	
Change in wound care	20.0% (N=5)
Earlier clinic appointment	16.0% (N=4)
Mean wound area at study completion, cm² (SD)	10.8 (11.9)
Mean wound area change from enrollment to completion, cm² (SD)	7.67 (9.72)
Wound healed at conclusion of study	12.0% (N=3)

Thirty-six percent of patients were advised that they should undergo an early change in their wound management plan at least once during the study based on weekly review of their wound scan by the study team. Treatment changes included a change in wound treatment in 20.0% of patients and initiation of an earlier appointment for in-person clinic evaluation in 16.0% of patients. There were no instances where use of the digital management system resulted in a delayed diagnosis of wound deterioration.

At the conclusion of the study, there was a mean decrease in wound area of 7.67 cm^2^ (SD, 9.72) per patient (P=0.005), equivalent to a mean decrease of 41.6% (SD, 15.8%). Complete wound healing was achieved in 12.0% of patients (3/25).

### Patient satisfaction

**Figure 3:**
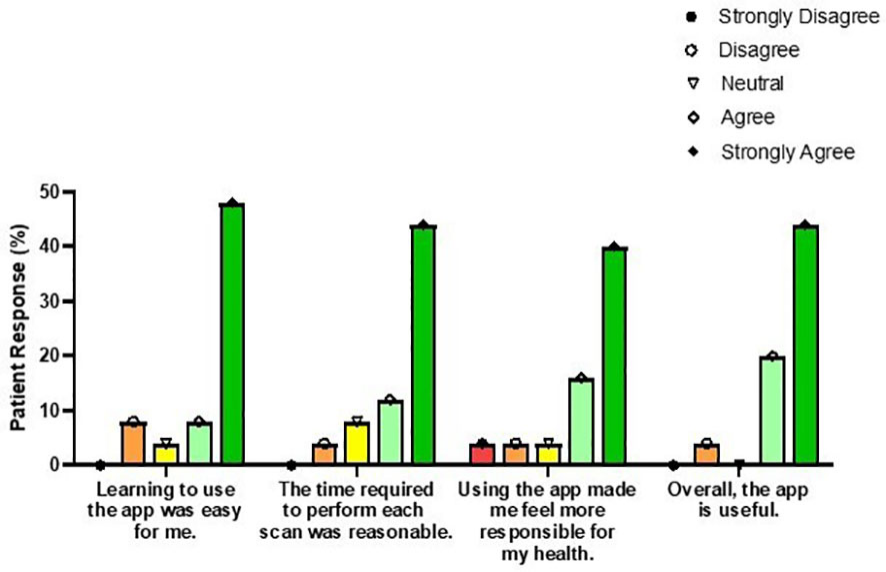
Post-study primary user survey results summarizing patient satisfaction with the Healthy.io Minuteful for Wound smartphone app.

Primary users who completed at least one at-home wound scan during the study period were asked to take part in a post-study survey, with a response rate of 81.0% (17/21). Of the four participants who did not complete the survey, three were unable to be reached by telephone after completion of the study and one declined to participate. Of the survey respondents, 88.2% agreed or strongly agreed that the Minuteful Wound app was easy to use. Overall, 94.1% of patients found the digital wound management system to be useful, with the large majority noting they felt more involved in their wound care, more responsible for their health, and more able to access healthcare services (**Figure 3**). One patient even stated that they felt “empowered [to be accountable for their health].”

Common themes that recurred throughout the patient surveys included appreciation for close wound monitoring without the need for travel to the clinic. However, many respondents expressed room for improvement with “more instantaneous feedback.” Despite the asynchronous design of the app use and study team evaluation, 100% of patients felt confident that the information they sent to the study team was received.

### Usage data outcomes

**Figure 4:**
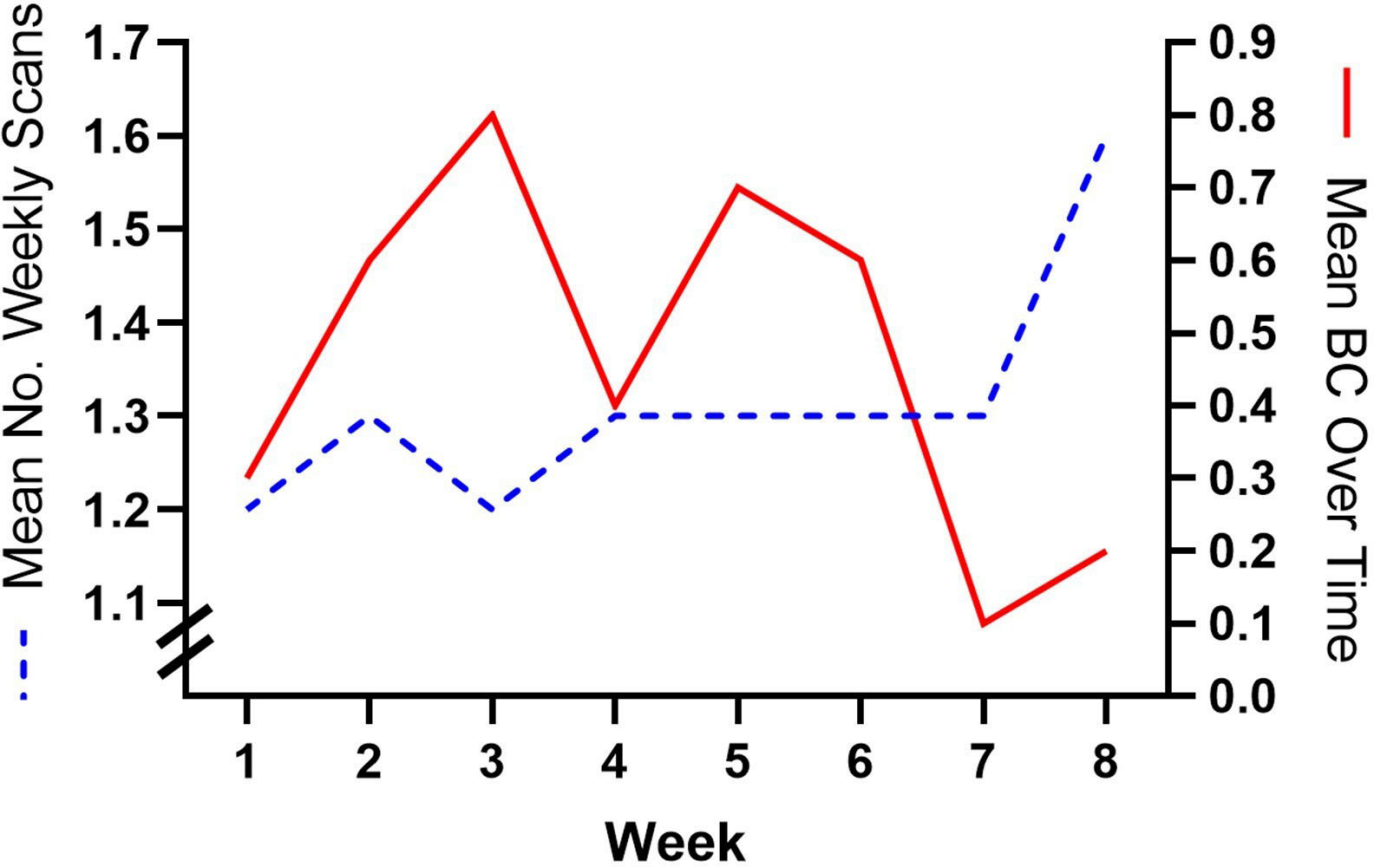
Mean number of weekly scans submitted (dotted blue line) and mean number of boundary condition notifications received (solid red line) by the Healthy.io Minuteful for Wound Digital Management System over the 8-week study period.

Among the 179 wound scans attempted by patients and/or their caregiver, 145 (81.0%) were free of boundary condition violations and successfully submitted to the Portal *via* the app. Eleven patients did not receive any boundary condition notifications during the study period, meaning they produced satisfactory and clinically valid wound documentation on all first wound scan attempts throughout the study period. Of the 34 scans that did not meet the prerequisite boundary conditions, 19 (55.9%) were rectified after a single user notification. The boundary conditions that were violated for these patients were much more common during the beginning of the study and decreased over the course of 8 weeks (**Figure 4**).

## Discussion

As our healthcare system has been adapting to the ever-changing climate of the COVID-19 pandemic, telemedicine advancement has become a priority for medical technology companies. We aimed to determine whether a novel digital wound monitoring system could be effectively used by patients and/or their caregivers to provide clinicians with high-quality wound data to guide care. We found that patients and caregivers could successfully learn how to use the Minuteful for Wound smartphone app, and the majority successfully engaged in its use. Study participants found the digital wound management system useful, and more than a third of patients benefited from its use in the form of early treatment modification.

A number of software companies have developed smartphone apps to measure and record wound size, including for DFU (15, 21–25). Remote wound monitoring programs may be helpful in the management of chronic wounds, particularly as a communication tool between patients and their healthcare providers when close follow-up is necessary (26). However, most of the applications are developed with physicians and nurses being the intended users. A recent study enrolled patients from a rural Veteran’s Affairs wound care clinic in a remote wound telemedicine program and showed excellent wound healing outcomes, but the wound telemedicine was facilitated by a trained telepresenter (27). Similarly, a meta-analysis of telemedicine versus in-person management of DFU showed similar or possibly improved wound healing, amputation, and mortality outcomes for patients managed *via* telemedicine (13). However, all telemedicine studies identified in the meta-analysis involved use of a trained nurse or similar healthcare provider to facilitate the telemedicine communication between the patient and physician. Our study is unique in that it assessed a remote wound monitoring system designed to be patient-facing, where patients and their caregivers had total responsibility for capturing and submitting remote wound scans on a repeated basis.

Patient engagement in our study was high compared to prior studies of telemedicine use. In a study of emergency department patients undergoing acute laceration repair or incision and drainage procedures, 58% of patients sent at least one picture of their wound through a Mobile Post-operative Wound Evaluator (mPOWEr) smartphone app (25). In our study, 84.0% of patients submitted at least one at-home wound scan. However, overall engagement was lower because we defined patient engagement as capturing ≥50% of expected weekly scans. The need for repeated wound scans places a larger burden on the patients and their caregivers than a single wound capture but was designed to simulate the frequency of standard in-person wound monitoring in the clinic. Reminder phone calls from the study team were required approximately twice per patient over the course of the 8-week study. Whether this burden is sustainable for larger numbers of patients is unclear. A prior study also demonstrated that telemedicine costs for managing DFU patients by telemedicine are approximately $2222 USD lower per patient compared to standard in-person monitoring (28).

Primary users in our study reported high rates of satisfaction with the Healthy.io Minuteful for Wound Digital Management System. Patients are generally in favor of remote wound monitoring based on data from prior studies, including studies specific to DFU (29–31). In a scoping review of telemedicine solutions for DFU, four main maps emerged: “A whole human not merely a hole in a human,” “Less of a burden on the family, the community, and the environment,” “Competences and continuity of care are essential for high-quality care” and “The quality and modality of the technology.” Consistent with these concepts, our patients reported less frequent in-person appointments, better continuity of care, and more accountability with care as benefits. We also observed some drawbacks to the technology. Specifically, primary users felt that more instantaneous feedback about the wound would be helpful. Future iterations of the app will involve a 2-way in-app communication tool that will allow patients to receive feedback more synchronously and remove the burden of the weekly phone call.

In addition to patients’ desire for more timely wound feedback, we encountered other challenges in our study. While our rate of study completion was high (72.0%), seven patients failed to complete the study. One of the major concerns around the use of remote wound monitoring systems is how they can be utilized by socioeconomically disadvantaged patients. Nearly one third of patients in our study required a borrowed smartphone because they lacked a smartphone with the specifications needed to run the app; most patients in our study had a preexisting smartphone, but many were older models not compatible with this technology. Both smartphone and reliable internet access are barriers to implementation in vulnerable populations (13, 32). Our multidisciplinary diabetic limb preservation clinic serves a large number of patients from socially disadvantaged backgrounds, however, patients enrolled in this study resided in less disadvantaged neighborhoods than a typical patient in our clinic (33). Making remote wound monitoring technology accessible to a wide range of populations will be important for successful adoption moving forward.

There are a number of limitations to this study. We enrolled only a small number of patients in this pilot study, and we did not have a control group for comparison. We were not able to evaluate hospital financial data associated with the implementation and continued use of the app, but plan to do so in the future. Finally, our study was not designed or powered to assess wound healing outcomes, and due to the feasibility design we did not attempt to alter patient care based on the wound images provided. However, our findings did lead to the successful initiation of a now ongoing randomized controlled trial comparing use of the Minuteful for Wound Digital Management System compared to standard of care in-person monitoring (34).

## Conclusion

The Healthy.io Minuteful for Wound Digital Management System is a feasible means of remote wound monitoring for use by patients and their caregivers. We were able to show good patient engagement, satisfaction, and usage data in a pilot study design. Our results suggest the feasibility of patient-facing technology for the remote wound app monitoring of diabetic foot ulcers. This study is the impetus for a new randomized controlled trial designed to study wound healing efficacy for remote wound app monitoring vs. standard in-person clinic visits for the treatment of lower extremity wounds, in which we hope to show the barriers that often interfere with in-person follow up visits will no longer interfere with proper wound care.

## Data availability statement

The raw data supporting the conclusions of this article will be made available by the authors, without undue reservation.

## Ethics statement

The studies involving human participants were reviewed and approved by Johns Hopkins University Institutional Review Board. The patients/participants provided their written informed consent to participate in this study. Written informed consent was obtained from the individual(s) for the publication of any potentially identifiable images or data included in this article.

## Author contributions

AK and CH, project conceptualization, patient enrollment, trial coordination, wound scan review, data analysis, manuscript development, and review. SB and EL-A, data analysis, manuscript development, and review. KM, MS, DS, CA, and ES, manuscript development and review. DJ, project conceptualization, data analysis, manuscript development, and review. RS, patient enrollment, wound scan review, manuscript development, and review. JR, patient enrollment and trial coordination. All authors contributed to the article and approved the submitted version.
