# Corrigendum: Implementation of a patient-centered remote wound monitoring system for management of diabetic foot ulcers

**DOI:** 10.3389/fendo.2023.1235970

**Published:** 2023-06-23

**Authors:** Alana C. Keegan, Sanuja Bose, Katherine M. McDermott, Midori P. Starks White, David P. Stonko, Danielle Jeddah, Eilat Lev-Ari, Joanna Rutkowski, Ronald Sherman, Christopher J. Abularrage, Elizabeth Selvin, Caitlin W. Hicks

**Affiliations:** ^1^ Department of Surgery, Sinai Hospital of Baltimore, Baltimore, MD, United States; ^2^ Division of Vascular Surgery and Endovascular Therapy, Johns Hopkins University, Baltimore, MD, United States; ^3^ Department of Clinical Development, Healthy.io Ltd., Tel Aviv, Israel; ^4^ Department of Epidemiology, Johns Hopkins School of Public Health, Baltimore, MD, United States

**Keywords:** diabetic foot ulcer (DFU), diabetes, smartphone application (app), telemedicine, technology, smartphone, diabetic foot

In the published article, there was an error in **Table 2** as published. Some of the characteristics that represent means (standard deviation) are designated as representing (%). The corrected **Table 2** and its caption appear below.

**Table 2:** Baseline wound characteristics and related surgical procedures of the study population.

Characteristic	Overall % (N=25)
Wound location	
Lateral/forefoot	44.0% (11)
Lower leg/ankle	32.0% (8)
Heel	12.0% (3)
Plantar foot	8.00% (2)
Toe	4.00% (1)
Wound area, mean cm² ± SD	18.0 ± 15.2
Osteomyelitis	24.0% (6)
WIfI Classification	
1	24.0% (6)
2	40.0% (10)
3	28.0% (7)
4	8.00% (2)
Toe pressure, mean mmHg ± SD (N= 17)	79.6 ± 45.0
Ankle Brachial Index, mean ± SD (N= 16)	1.0 ± 0.2
Related revascularization procedure	
Endovascular	32.0% (8)
Open	32.0% (8)
No. revascularization procedures, mean ± SD	1.1 ± 1.5
Podiatric interventions of the affected limb*	
None	20.0% (5)
Bone resection/debridement	56.0% (14)
Biologic coverage	48.0% (12)
Minor amputation	40.0% (10)
Skin graft	16.0% (4)
Home care	60.0% (15)
Wound dressing type	
Collagen	24.0% (6)
Negative pressure wound therapy	16.0% (4)
Wound hydration	24.0% (6)
Enzymatic debridement	12.0% (3)
Filler	24.0% (6)

WIfI, Wound, Ischemia, and foot Infection.

*Sum equals greater than 100% because some patients received more than one podiatric intervention.

The authors apologize for this error and state that this does not change the scientific conclusions of the article in any way. The original article has been updated.

