# Supplementary material for: Implementation of a patient-centered remote wound monitoring system for management of diabetic foot ulcers
Source: Front Endocrinol (Lausanne). 2023 May 24;14:1157518. doi: 10.3389/fendo.2023.1157518 (PMC10244728; doi:10.3389/fendo.2023.1157518)
Supplement: Supplementary file 1 [file Table_1.docx]

**Implementation of a Patient-Centered Remote Wound Monitoring System for Management of Diabetic Foot Ulcers**

Alana C. Keegan MD¹ ², Sanuja Bose MD, MPH², Katherine M. McDermott MD², Midori P. Starks WhiteMD², David P. Stonko MD, MS², Danielle Jeddah MD³, Eilat Lev-Ari³, Joanna Rutkowski RN, BSN², Ronald Sherman DPM², Christopher J. Abularrage MD², Elizabeth Selvin PhD, MPH^4^, Caitlin W. Hicks, MD, MS²

¹ Department of Surgery, Sinai Hospital of Baltimore, Baltimore, MD

² Division of Vascular Surgery and Endovascular Therapy, Johns Hopkins University, Baltimore, MD

³ Healthy.io Ltd, Tel Aviv, Israel

^4^ Department of Epidemiology, Johns Hopkins School of Public Health, Baltimore, MD

**Corresponding Author:**

Caitlin W. Hicks MD, MS

Division of Vascular Surgery and Endovascular Therapy

Johns Hopkins University

600 N Wolfe Street, Halsted 668

Baltimore, MD 21287

Email: chicks11@jhmi.edu

**
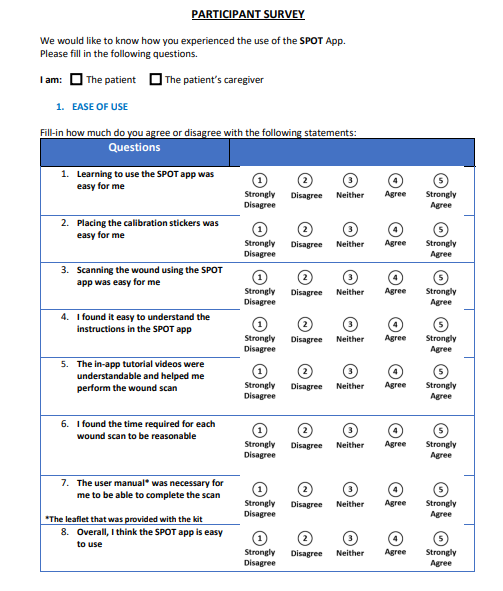
Supplementary Table 1.**

**
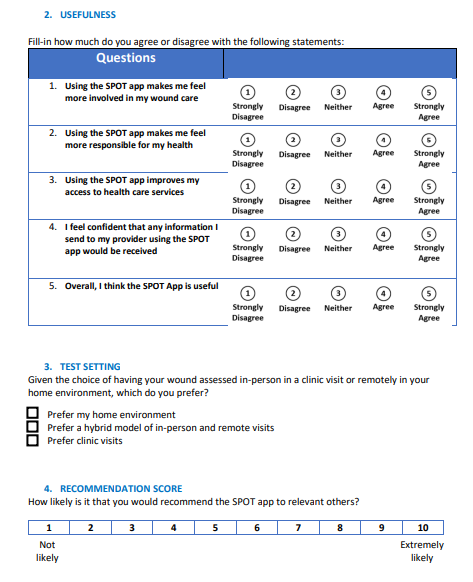
**

**
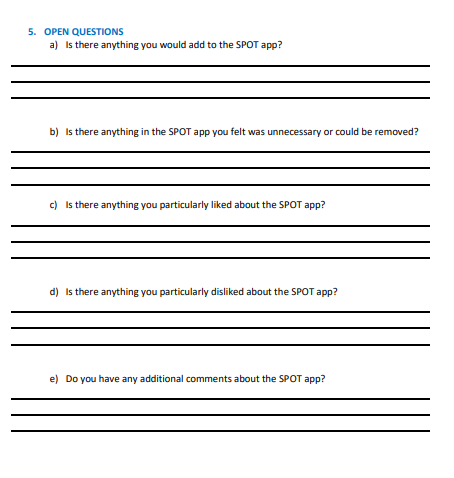
**
